# The Role of IL-37 and IL-38 in Obstetrics Abnormalities

**DOI:** 10.3389/fmed.2021.737084

**Published:** 2021-08-27

**Authors:** Mei Wang

**Affiliations:** Department of Obstetrics and Gynaecology, Gansu Provincial Hospital, Lanzhou, China

**Keywords:** gestational diabetes mellitus, pre-eclampsia, IL-37, IL-38, obstetrics

## Abstract

There are two fairly common complications during pregnancy, i.e., gestational diabetes mellitus (GDM) and pre-eclampsia, which are independent, but are also closely linked in prevalence in pregnant women, with potential serious adverse consequences. IL-37 and IL-38, which belong to the IL-1 superfamily, participate in anti-inflammatory responses. Dysregulation of IL-37 and IL-38 has been observed in many auto-immune diseases. IL-37 is substantially reduced in the umbilical cords and placentas of GDM subjects, but IL-37 is significantly induced in the placentas of pre-eclampsia patients, suggesting there are differential regulatory roles of IL-37 in obstetrics, despite IL-37 being an anti-inflammatory mediator. Furthermore, IL-38 is substantially increased in the umbilical cords and placentas of GDM subjects, but minimal difference is observed in the placentas from pre-eclampsia patients. These data imply that IL-38 is also regulated independently within the diseased placentas. This review provides some insight for both basic scientists and medical practitioners to manage these patients effectively.

## Introduction

The aim is to illustrate involvement of dysregulated IL-37 and IL-38 in gestational diabetes mellitus (GDM) and pre-eclampsia, which are two rather common and closely linked complications during pregnancy (1–3), i.e., there is a substantially high prevalence of pre-eclampsia among women with hyperglycaemia either short term (GDM) or long term (DM). Notably, both of these conditions either result in and/or from pathophysiological changes within the placenta during pregnancy, with substantial immunopathological involvement. Previous reviews have assessed the involvement of proinflammatory cytokines (IL-1, TNF, IL-18) (4), and IL-34 (5) in GMD. In the pre- eclampsia women there are large amounts of both pro-inflammatory and anti-inflammatory mediators released from the placenta, e.g., IL-1, IL-10, IL-18, IL-36s, IL-37, and IL-38 (6). However, the involvement of dysregulated IL-37 and IL-38 in GDM and pre-eclampsia has not been reviewed yet. Two relatively novel cytokines, i.e., IL-37 and IL-38, are considered to play critical roles during host immunological responses. Therefore, this review focuses on possible roles of IL-37/IL-38 in GDM and pre-eclampsia.

GDM is associated with adverse maternal health outcomes, including pre-eclampsia and neonatal problems, as well as a high risk of the development of obesity and type 2 diabetes mellitus (7). GDM is defined as hyperglycaemia during the gestational period in women without a previous history of diabetes (8). More specifically GDM occurs during pregnancy commonly at 24–28 weeks, due to insulin resistance causing higher than normal blood glucose levels during pregnancy. Increased blood glucose is a normal compensatory response observed in pregnant women that ensures a sufficient glucose supply to the fetus for development (9). However, uncontrolled hyperglycaemia during pregnancy is partially due to decreased insulin sensitivity by almost 60%. Furthermore, hyperglycaemia is exacerbated by the production of insulin antagonists i.e., human chorionic gonadotropin and prolactin during pregnancy (10), which reach their physiological peaked from 8 weeks gestation onwards (11). In addition, there is ~50% increase in the size of β cells in in the pancreatic islets from GDM pregnant women (10) as a compensation for the gestational hyperglycaemia. The most serious concerns for uncontrolled high blood glucose during GDM would be malignant hypertension and pre-eclampsia, often associated with epileptic seizures, which usually requires immediate cesarean section for the protection of both the mother and baby. The long-term consequences of GDM would be the risk of developing type 2 diabetes for GDM mothers (1, 2). In addition to long term morbidity for women suffering from GDM, there is a significantly increased risk of development of diabetes and obesity in the infants delivered from GDM mothers during childhood and adulthood (2).

The underlying mechanism (pathophysiology) of GDM is probably related to substantial over-production of placenta-produced hormones (prolactin and human chorionic gonadotropin) with reduced tolerance in some susceptible pregnant women (10). There is a close correlation between systemic/local inflammation and diabetes mellitus (12), accompanied with significantly increased proinflammatory cytokines, e.g., IL-1β, IL-6, IL-17, reactive protein and TNF. Consequently, the substantially increased inflammatory mediators are capable of further interfering with the insulin signal pathway, causing more insulin resistance (13). Thus, induced systemic and local inflammation following uncontrolled hyperglycaemia is characteristic of the placenta in GDM.

As mentioned above, pre-eclampsia could be caused or exacerbated by GDM, and represents another abnormality that contributes to maternal systemic inflammation (14).

It is defined by The American College of Obstetrics and Gynaecology that pre-eclampsia is a disorder of pregnancy associated with new-onset hypertension, which occurs most often after 20 weeks of gestation and frequently near term. Although often accompanied by new-onset proteinuria, hypertension and other signs or symptoms of pre-eclampsia may present in some women in the absence of proteinuria (15). The more detailed diagnostic criteria for pre-eclampsia have been well-documented (15), and will not be presented in the current review.

Delivery can resolve most signs and symptoms of GDM and pre-eclampsia usually. Pre-eclampsia, however, could persist even after delivery. It has been reported that it could be a great risk factor contributing to cardiovascular disease and cerebrovascular accidents in preterm pre-eclampsia (16).

Pre-eclampsia presents clinically with uncontrolled maternal hypertension and proteinuria during the second half of pregnancy onwards, or chronic hypertension with superimposed preeclampsia - chronic hypertension with new-onset proteinuria or other signs/symptoms of preeclampsia with morbidity occurring in up to 8% of all pregnancies (14). The pathogenesis of pre-eclampsia is due to dysfunctional uteroplacental perfusion, causing hypoxia and oxidative stress and subsequently damaging the placenta during the early stages of pregnancy (17). The main reason for dysfunction of uteroplacental perfusion is because of abnormality of the trophoblasts from the placenta creating/releasing of pro-inflammatory mediators, causing local (placental) and systemic inflammations (18). It is also understandable that secretion of these factors can be detected during normal pregnancy, showing mild systemic inflammation increases as pregnancy progresses, however, the levels of such factors are not as high as in the abnormal pre-eclampsia individuals. Despite this mild inflammation, the immunologically foreign, semi-allogeneic fetus is tolerated by maternal immune cells. In the pre- eclampsia women there are large amounts of both pro-inflammatory and anti-inflammatory mediators released from the placenta, e.g., IL-1, IL-10, IL-18, IL-36s, IL-37, and IL-38 (6).

## IL-37

IL-37, which belongs to IL-1 superfamily, was originally named as IL-1 family member 7 (IL-1F7), because it is the seventh member of the IL-1 family discovered (19) and shares the structural pattern of the IL-1 family (20). The IL-37 gene size is 3.617 kb, with six exons, and encodes a 17–26 KDa protein (21). There is a constitutive level of IL-37 production in normal cells (including NK cells, activated B cells, monocytes, keratinocytes and epithelial cells) and tissues (e.g., lymph node, thymus, lung, intestine and uterus) (22, 23). The biological function of IL-37 is considered to be an anti-inflammatory cytokine, because IL-37 is able to supress innate (20) and adaptive immunity (24), which can explain the consequent reduction of the host response (25), including in tumorigenesis (26). The anti-inflammatory function of IL-37 (25) is partially due to the capacity of IL-37 to inhibit the maturation of dendritic cells through the IL-1R8-TLR4-NF-κB pathway (27). In addition, it has been reported that an anti-inflammatory role of IL-37 is observed in autoimmune diseases, such as rheumatoid arthritis, psoriasis, Grave's disease and systemic lupus erythematous, and in ulcerative colitis and Crohn's disease, perhaps *via* inhibiting the expression and function of pro-inflammatory cytokines (28–30). In addition, IL-37 substantially reduces the development of atherosclerosis in an IL-37 transgenic animal model (31).

## IL-38

IL-38, which also belongs to the IL-1 family, was originally discovered as a novel member of the human IL-1 gene family (IL1HY1), with 41% homology with IL-1Ra and 43% homology with IL-36R (32). The expression of IL-38 has been demonstrated in embryonic tissues, as well as in adult tissues, e.g., cardiovascular, respiratory, gastrointestinal, reproductive systems, and cutaneous epithelium, brain, liver etc. (33). IL-38 is constitutively expressed in very low amounts in many tissues and has an anti-inflammatory role (34) for maintaining homeostasis within the micro-environment. The anti-inflammatory role of IL-38 includes the release of IL-38 from apoptotic cells to limit inflammatory macrophage responses (35). Thus, any disturbance of IL-38 can cause mal-adaptive clinical responses. From a functionality point of view, IL-38 displaces inflammatory signaling, analogous to the IL-1ab/receptor agonist and IL-1R1, as an anti-inflammatory mediator. Dysregulation of IL-38 can initiate host immunity due to an imbalance of the pro- vs. anti-inflammatory micro-environment, causing inflammatory diseases. The mRNA expression of IL-38 is up-regulated in inflamed skin (33). However, a significant reduction of IL-38 is detected in psoriatic skin (36). IL-38 is supressed in psoriatic skin in response to the proinflammatory cytokines IL-36γ, IL-17, and IL-22n (37). Recombinant IL-38 counteracts the biological processes induced by IL-36γ in epithelial and endothelial cells and exogenous IL-38 attenuates the severity of psoriasis. In addition, IL-38 is increased in the infiltrating leucocytes and epithelial cells of active inflammatory bowel disease patients compared to that of control (38), and also in rheumatoid arthritis (39), coronary ischemic disease (40) and an experimental liver injury model (41).

## IL-37 in Obstetrics

### IL-37 in GDM

A constitutive level of expression of IL-37 is detected in the chorionic villi and umbilical cords of normal pregnant women, but importantly IL-37 is reduced by almost 50% in the intima of the arteries from GDM women. Although GDM is a relatively short-term form of pathophysiological hyperglycaemia in pregnant women, associated with an highly inflammatory status in the micro-environment (12), reduced IL-37 may not be able to provide anti-inflammatory protection in both local (placental) and systemic responses, and consequently high levels of inflammation are induced in GDM pregnant women. Such a finding is in line with others, showing that there are significantly reduced levels of both circulating and local (chorionic villi) of an anti-inflammatory mediator (gal-13) in GDM (42). It has been reported that there is an inverse correlation between IL-37 and inflammatory cytokines (43), perhaps due to the capacity of IL-37 to inhibit reactive oxygen species and pro-inflammatory cytokines (44).

Thus, reduced IL-37 in GDM pregnant women may compromise the protection from inflammation for the umbilical cord and chorionic villi, resulting in even higher inflammation in the placenta, mediated in both an autocrine and paracrine fashion, perhaps *via* supressing both innate and acquired immune responses (20, 24). In addition to direct regulation of the immune system, IL-37 is also correlated with insulin sensitivity (43), which could contribute to the uncontrolled hyperglycaemia status in GDM women. On the other hand, a constitutive level of expression of IL-37 in the placenta from Non-GDM pregnant women is most likely protecting the healthy umbilical cord and chorionic villi (45). This observation is consistent with significantly more neo-vascularisation in the placental chorionic villi, as well as increased thickened of the intima in the umbilical arteries from GDM compared to non-GDM women (45). The increased intimal thickness may be due to the transition to the diabetic micro-environment during pregnancy, in line with increased atheroma in diabetic patients (12). However, no significant difference in IL-37 expression is observed in the umbilical cord interstitial tissues between GDM and Non-GDM subjects, suggesting that diabetic-mediated vascular change is less effectively modified by IL-37 (45), perhaps due to too short a GDM time period.

The precise pathogenesis of IL-37 involved in GDM requires further clarification, although the observation that reduced systemic and local IL-37 may contribute to the development of GDM appears to be established.

### IL-37 in Pre-eclampsia

Pre-eclampsia is another serious condition during pregnancy. Interestingly, the production of IL-37 has been shown to be increased more than 5-fold in the placentas from pre-eclampsia compared to that of normal pregnancies at the protein level (6). Pre-eclampsia is another physiopathological condition, that is mainly due to damage to the placenta trophoblasts (17). Increased IL-37 correlates with elevated pro-inflammatory cytokine production, i.e., IL-18, IL-36 α, β, γ, as well as increased IL-36Rα in the placenta (6). IL-37 is an anti-inflammatory cytokine; whereas the proinflammatory role of IL-18 and the IL-36s are well-documented (46–48). Thus, upregulated IL-37 in the placenta during pre-eclampsia is probably a compensatory response toward the high level of pro-inflammatory stimuli (21), but the anti-inflammatory effect of IL-37 perhaps is insufficient to quench such a large amount of inflammatory mediators in susceptible individuals. Interestingly, there is no significant difference in circulating IL-37 between pre-eclampsia and normal pregnancy (6), implying that the challenge of pre-eclampsia is more localized in the placenta, rather than systemic, although pre-eclampsia is a more systemic condition during pregnancy (17). It is understandable that pre-eclampsia is a short-term pathophysiological condition, which is usually resolved once the pregnancy is completed (14). This is supported by evidence that sudden seizure in pre-eclampsia patients with uncontrolled hypertension requires immediately termination of the pregnancy with cesarean section in the pre-eclampsia pregnancy without other co-existing conditions following forced cesarean (49).

## IL-38 in Obstetrics

There is constitutive production of IL-38 in the chorionic villi of the placenta and the umbilical cord in normal pregnant women, supporting the hypothesis that IL-38 contributes to inflammatory balance and/or homeostasis (34, 37, 50, 51). This is consistent with the finding that IL-38 acts as an antagonist for IL-36, inhibiting the inflammatory response (37). However, substantially upregulated IL-38 is detected in the umbilical artery intima in the umbilical cord from GDM women. Since it is well-understood that IL-38 is an anti-inflammatory cytokine, increased IL-38 in GDM probably contributes to the suppression of the highly inflamed placenta and umbilical cord during the GDM condition, which is in line with significantly upregulated proinflammatory cytokines (IL-6 and TNF) in the GDM placenta (45), and the higher levels of systemic and local proinflammatory cytokines in the diabetic condition (12). Although upregulated IL-38 has some suppressive effects under normal conditions, that may be a consequence of mild uncontrolled hyperglycaemia (52), such an anti-inflammatory effect is likely insufficient for quenching the local environment during the GDM condition in susceptible subjects (1, 2, 9). Thus, it is fundamentally important to strictly control the blood glucose level by all means to minimize potential and irreversible adverse consequence(s). Although GDM is a relatively short-term abnormality, i.e., up to 40 weeks duration, systemic and local inflammation is still highly induced. The consequence of hyperglycaemia may not be as serious as conventional diabetes mellitus, with long term disease progression. This is in line with the finding that there is just detectable IL-38 expression in the interstitial tissues of the umbilical cord from both GDM and non-GDM subjects, but no significant elevation is observed between these two groups (45).

IL-38 production is detected in the placenta from pre-eclampsia and normal pregnant subjects, showing no significant difference (6). However, there is substantial upregulation of IL-37 in the placentas from pre-eclampsia subjects. In addition, there is differential regulation of IL-37 and IL-38 in the placenta from GDM subjects (45), which parallels the observations from colorectal cancers (34, 53, 54), thus inviting speculation that IL-38 would be also be regulated to participate in the local immunopathological-mediated condition in pre-eclampsia subjects for reducing the immune response, perhaps *via* releasing IL-38 from apoptotic cells to limit inflammatory macrophage responses (35).

In conclusion, IL-37 and IL-38 are two key immune regulators in the anti-inflammatory responses that protect pregnant women. However, dysregulation of IL-37 and IL-38 in GDM or pre-eclampsia, probably plays different regulatory roles with rather different outcomes, although both of these two conditions relate to obstetric abnormalities. Understanding the involvement of IL-37 and IL-38 in the pathogenesis of GDM or pre-eclampsia may be a milestone in the field of obstetrics.

Furthermore, the observations that IL-37 and IL-38, anti-inflammatory cytokines, are dysregulated in the placenta during the progression of GDM and pre-eclampsia, invites speculation that IL-37 and/or IL-38 could be target(s) in preventing and/or management of these two obstetric abnormalities, eventually reducing local and/or systemic inflammation. These data thus provide some insight for the development of potential therapeutic target(s) and/or pharmacological pathways in the management of obstetric abnormalities.

## Author Contributions

The author confirms being the sole contributor of this work and has approved it for publication.

## Conflict of Interest

The author declares that the research was conducted in the absence of any commercial or financial relationships that could be construed as a potential conflict of interest.

## Publisher's Note

All claims expressed in this article are solely those of the authors and do not necessarily represent those of their affiliated organizations, or those of the publisher, the editors and the reviewers. Any product that may be evaluated in this article, or claim that may be made by its manufacturer, is not guaranteed or endorsed by the publisher.
